# Supplementary material for: Increased Risk of Developing Depression in Disability after Stroke: A Korean Nationwide Study
Source: Int J Environ Res Public Health. 2023 Jan 2;20(1):842. doi: 10.3390/ijerph20010842 (PMC9819798; doi:10.3390/ijerph20010842)
Supplement: Supplementary file 1 [file ijerph-20-00842-s001.zip › ijerph-2116004-supplementary.pdf]

Table S1. Definition of severity of disability after brain injury

Grade	Definitions
1	Unable to walk independently, and totally requires assistance from others<br>Unable to carry on any ordinary activities due to complete paralysis of both arms, and totally requires assistance from others<br>Unable to carry on any ordinary activities due to complete paralysis of one arm and one leg, and totally requires assistance from others<br>The Modified Barthel Index is less or equal to 32 points, and totally requires assistance from others to perform all ordinary activities including ambulation
2	Unable to carry on any ordinary activities due to complete paralysis of one arm, and totally requires assistance from others<br>Unable to use all fingers of both hands due to complete paralysis and joint contracture, and totally requires assistance from others<br>The Modified Barthel Index is between 33 and 53 points, and requires assistance from others to perform most of ordinary activities including ambulation
3	Unable to use all fingers of one hand due to complete paralysis and joint contracture, and totally requires assistance from others<br>Unable to walk due to complete paralysis of one leg, and mostly requires assistance from others<br>The Modified Barthel Index is between 54 and 69 points, and unable to perform ordinary activities independently and requires partial assistance from others
4	The Modified Barthel Index is between 70 and 80 points, and able to perform ordinary activities but intermittently requires assistance from others
5	The Modified Barthel Index is between 81 and 89 points, and able to perform most of ordinary activities independently but sometimes requires assistance from others
6	The Modified Barthel Index is between 81 and 89 points, and able to perform most of ordinary activities independently but sometimes requires more time
